# The Double-Opposing Adipofascial Interposition Flap: A Novel Technique to Prevent Scar Tethering and Symptomatic Neuroma of the Superficial Radial Nerve After Trauma

**DOI:** 10.7759/cureus.46081

**Published:** 2023-09-27

**Authors:** Chane Kulenkampff, Rajan Choudhary, Niall O'Hara, Samuel George

**Affiliations:** 1 Department of Plastic and Reconstructive Surgery, Queen Elizabeth Hospital, Birmingham, GBR

**Keywords:** nerve tether, nerve wrap, traumatic neuroma, adipofascial flap, superficial radial nerve

## Abstract

The superficial radial nerve (SRN) is vulnerable to injury following trauma with a high incidence of resultant nerve tether and neuroma formation. The SRN has an anatomical predisposition to neuroma formation, with research indicating that its propensity to neuroma development is out of proportion with its likelihood for injury. In addition, SRN neuromas have been described as one of the more painful and difficult neuromas to manage. Despite this, the published literature to date is chiefly focused on neuroma and scar tether treatment options rather than more impactful work on neuroma prevention, which can be safely delivered at the time of primary surgery. Treatment of established neuroma or nerve tether is notoriously difficult, and existing techniques have inconsistent outcomes, with patients often requiring multiple trips to the theatre. The authors present a novel technique for neuroma and scar tether prevention using an adipofascial flap accompanied by patient examples of our experience using this approach as an adjunct during the primary SRN repair, creating a gliding, interposing layer to prevent subsequent nerve traction pain and symptomatic neuroma.

We identified five patients presenting with dorsal wrist injuries involving the SRN and one or more tendons. Patients’ follow-up duration was a mean of 3.5 months (one to eight months). All follow-up patients showed no symptoms of a neuroma or nerve tether pain. All patients were discharged without re-referral or further surgery. Our patient sample demonstrates promising results using an adipofascial interposition flap as a prophylactic measure in traumatic injuries to reduce nerve tether pain and symptomatic neuroma formation in the SRN.

## Introduction

The superficial radial nerve (SRN) courses superficially in the distal forearm and is the third most injured peripheral nerve [1]. After branching off from the radial nerve at the level of the radio capitellar joint, it initially lies deep to the brachioradialis and pierces the deep fascia of the forearm approximately 9 cm proximal to the radial styloid. Distally, the nerve travels between the tendons of the brachioradialis and extensor carpi radialis longus [2]. Due to its anatomical location, the SRN is particularly vulnerable to damage in patients presenting with hand and wrist trauma. A significant sequelae of nerve injury is neuropathic pain, which may result following trauma or surgical repair. The pathological aetiology in these cases results from one of the following: formation of end neuromas, neuromas in-continuity, and nerve traction pain secondary to irritation, scar tether, or compression [3].

A neuroma of the SRN is one of the most common and most difficult neuromas to treat [4]. Newer techniques have begun looking at using nerve or vein wraps, as well as local, pedicled, or free flaps to cover and protect repaired or revised nerve segments [5]. It is thought that vascularised flaps provide nutrition and mechanical support to the nerve, favouring neurochemical and electrical stability with reduced irritability [6]. There is reduced scar adhesion to nearby structures, and the flap provides padding along the injured nerve from external forces and prevents the nerve from resting in a vulnerable position close to the skin while creating a gliding layer for the nerve. Fascial flaps offer further benefits due to their truly plastic nature and low friction coefficient, with improved nerve protection and regeneration [6-8].

To date, this technique is only described as a revision surgical procedure in cases of established nerve scarring or neuroma management. To the authors’ knowledge, there is no literature studying the use of such techniques during the primary operation as a prophylactic measure.

Study aim

The purpose of this study was to develop a novel surgical technique that could be used to minimise the risk of scarring between tendons and nerves in patients with traumatic injuries to the hand involving multiple structures. Ultimately, it is the hope that this technique may minimise the risk of post-operative nerve tether pain and symptomatic neuroma formation.

In this technical report, we describe a novel technique in a step-by-step fashion with accompanying diagrams and five illustrative cases in which this technique has been safely performed at the time of SRN repair as a prophylactic measure to prevent scar tethering and symptomatic neuroma.

This article was previously presented as a meeting abstract at the 2023 FESSH (Federation of European Societies for Surgery of the Hand) Conference on May 11, 2023, and the BAPRAS & NVPC Combined Meeting on June 16, 2023.

## Technical report

Between May 2019 and 2021, we identified five patients presenting with traumatic lacerations to the dorso-radial wrist involving the SRN and one or more neighbouring tendons. These patients had a double-opposing adipofascial interposition flap to protect the SRN after tendon and nerve repairs. Patients who needed nerve grafts were not included, and all nerves were repaired directly. All procedures were performed by the senior author and were performed with microscopic assistance under tourniquet control and brachial plexus anaesthesia.

**Figure 1:**
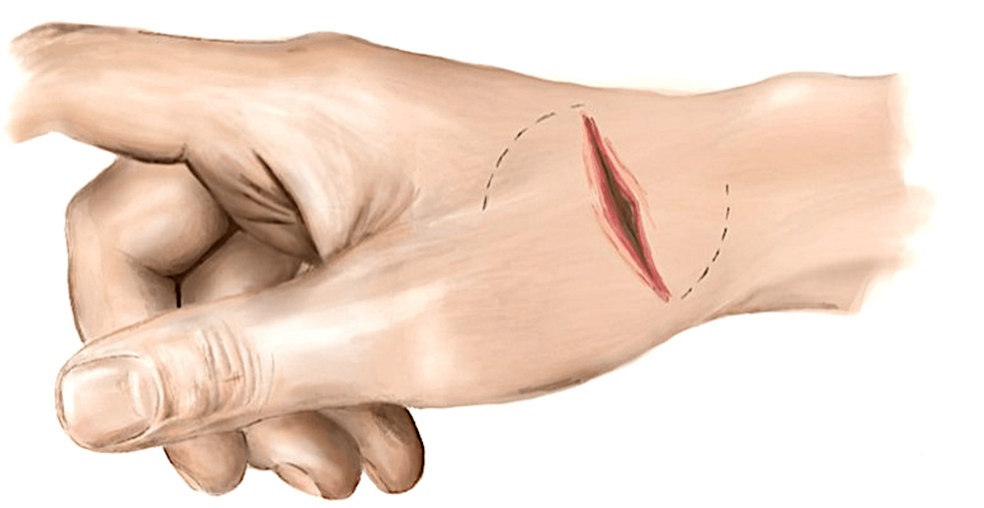
Illustration showing incision made to extend the dorsal-radial wound in a lazy S fashion along the axis of the SRN SRN, superficial radial nerve

**Figure 2:**
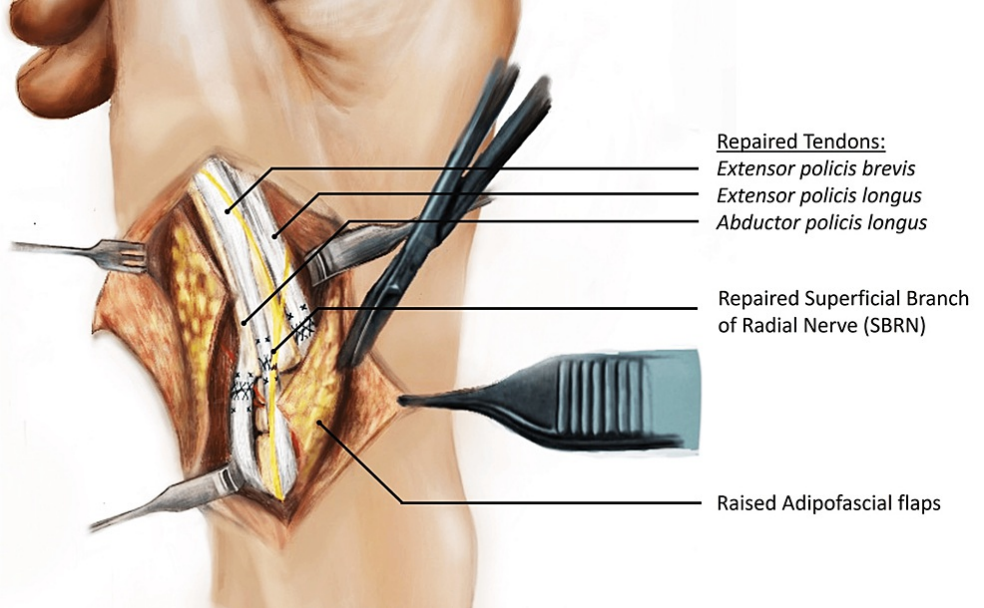
Illustration showing extended laceration showing tendon and SRN repair with adipofascial flaps meticulously lifted from overlying skin SRN, superficial radial nerve

**Figure 3:**
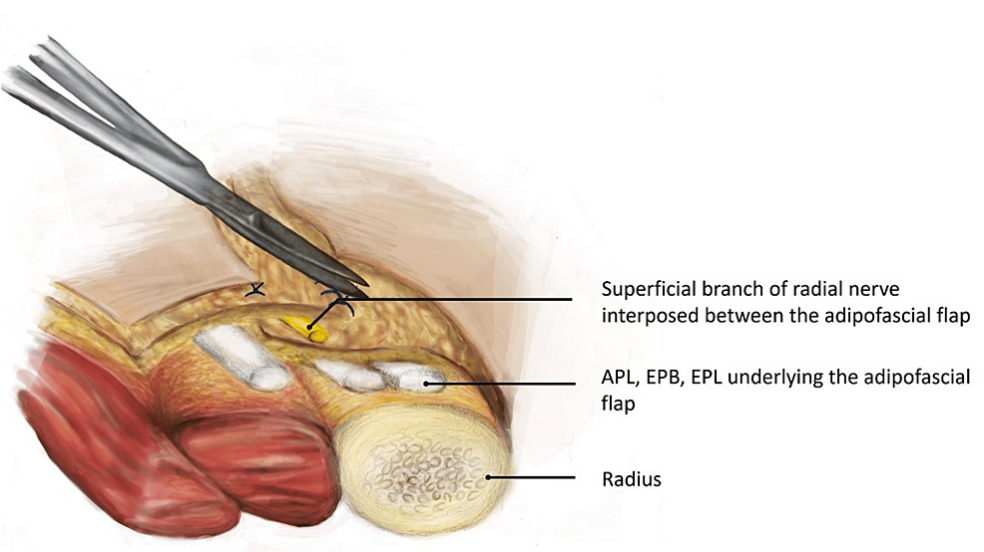
Illustration showing SRN interposed between the adipofascial flap, creating a plane between the SRN and underlying tendons that have undergone repair SRN, superficial radial nerve; EPB, extensor pollicis brevis; EPL, extensor pollicis longus

**Figure 4:**
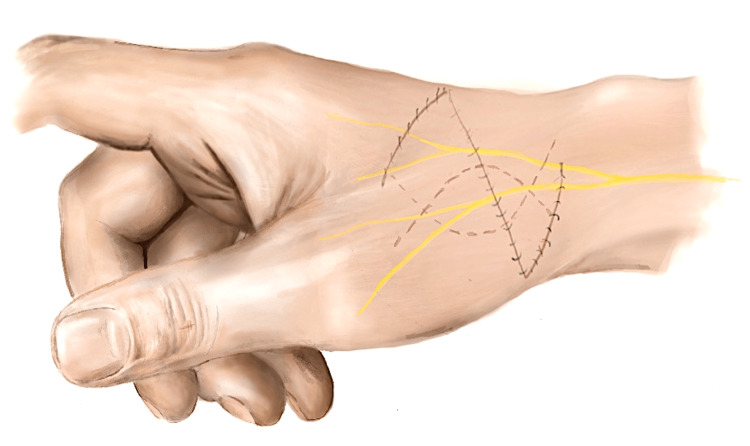
Illustration showing closed laceration with lazy S extensions underlying the double-opposing adipofascial flap, as well as the path of the SRN SRN, superficial radial nerve

An exploratory incision was made to extend the wound (usually a transverse dorsal-radial wound) in a lazy S or Z fashion distally and proximally along the axis of the SRN (Figure 1). The skin flaps were raised to include skin, subcutaneous tissue, and fascia. After tendon repairs were completed, two triangular adipofascial flaps were carefully separated from the skin flaps, being cautious not to excessively thin the skin flaps (Figure 2). These flaps were then interposed between the repaired tendons and nerves in a double-breasted fashion to effectively “wrap” the nerve in adipofascial tissue (Figure 3). The flaps were then anchored with 4-0 Vicryl Rapide. The resulting flap enveloped the nerve repair site to create a new non-irritant gliding plane that was separated from the healing tendons and the skin simultaneously (Figure 4). Post-operative immobilisation was tailored to the tendon repairs. Follow-up with the senior author and hand therapists was arranged at regular intervals for all patients.

Results 

All the patients were male, with a mean age of 35.8 (range 22-56). The clinical data of the patients are summarised in (Table 1). In our review, the most common mechanism of injury was glass laceration, which occurred in two of the five patients. Other mechanisms of injury included accidental angle grinder injury, accidental hand saw injury, and self-harm with a blade. One of the patients was a heavy smoker with COPD, and another had a background of schizophrenia. The remaining three patients were fit and well. All five patients had SRN injuries and associated tendon injuries. Extensor pollicis brevis (EPB) was injured in three patients, abductor pollicis longus (APL) was injured in three patients, extensor pollicis longus (EPL) was injured in two patients, and one patient additionally had extensor carpi radialis longus (ECRL) and brevis (ECRB) tendon injuries.

**Table 1:** Patient demographic and clinical data SRN, superficial radial nerve; COPD, chronic obstructive pulmonary disease; EPB, extensor pollicis brevis; APL, abductor pollicis longus; EPL, extensor pollicis longus; ECRL, extensor carpi radialis longus; ECRB, extensor carpi radialis brevis

Included patients	Five patients with traumatic lacerations to the dorsal wrist involving SRN plus ≥1 tendon
Demographics	All male patients, mean age: 35.8 (25-56)
Mechanism of injury	Glass lacerations (2), angle grinder (1), hand saw (1), and deliberate self-harm (1)
Comorbidities	Fit and well (3), COPD + heavy smoker (1), and schizophrenia (1)
Associated tendon injuries	EPB (3), APL (3), EPL (2), and ECRL and ECRB (1)

Patients attended with a mean follow-up of 3.5 months (range one to eight months, n = 4). All patients were scheduled for dressing and hand therapy follow-up one to two weeks post-op. Consultant-led clinic follow-up was scheduled at week six post-surgery. Of the five patients, one was lost to follow-up and two patients only attended dressing clinic and hand therapy follow-up. The remaining two patients attended consultant-led clinics at four- and six-months post-surgery, respectively. All patients that attended consultant follow-up showed no signs or symptoms of neuroma or nerve tether pain at the site of repair, and an advancing Tinel’s sign was documented at repeat examinations. All patients underwent hand physiotherapy and were discharged without complications. Complications sought with specific relation to the technique included reports of skin flap necrosis, surgical site infection, wound dehiscence or breakdown, and poor scar healing. No patients requested a further referral to the hand specialist team following discharge.

## Discussion

The pain from extra-neural scarring may itself result from several different mechanisms: impairment of epineural blood flow leading to nerve ischaemia, circumferential scarring causing mechanical compression of the nerve, and adhesions prohibiting gliding of the nerve leading to traction neuritis [9,10]. Traction neuritis may be further compounded if adjacent tendons or other neighbouring structures are injured, increasing the risk of adhesions to tendons or skin. It is hypothesised that the elimination of smooth gliding between structures, growth of sensitised axons and resultant tethering to neighbouring structures, and mechanical distortion of the nerve can cause it to fire spontaneously, resulting in neuropathic pain known as neurostenalgia [10]. This is further compounded by the pro-inflammatory environment that is thought to further sensitise nociceptive fibres to hyperalgesic responses [10].

SRN neuroma management depends on whether the neuroma is an end neuroma or in-continuity and if an active or passive reconstructive/ablative procedure is required [11]. For recurrent neuromas, neurectomy and passive ablative procedures are the mainstays of treatment in most units, but recurrence is an issue. Neural adhesions, though less well described than neuromas, can elicit nerve pain, particularly in the SRN, as the nerve is very prone to irritation and scarring [4]. Nerve pain appears one to three months after trauma or surgery following the inflammatory phase of recovery [10].

There have been an increasing number of techniques described for managing neuromas and neurostenalgia, with preferred approaches focusing on neurolysis, excision of neuromas, and relocation of the neural stump into proximal sites such as muscle or bone [3]. It is thought that neurolysis or relocation removes the irritant environment and reduces movement-induced nerve firing. Dellon and Mackinnon showed that burying nerves in muscle caused the formation of a smooth round bulb at the nerve end with organised and oriented fibres in parallel to each other [3]. This meant that the nerves were non-adherent to surrounding tissue and showed reduced excitability compared to their control group, which displayed disorganised fibres with a cap of fibrous tissue [3]. Unfortunately, using this method for the treatment of painful nerve in-continuity presents a problem as dividing or relocating nerves sacrifices continuity and distal nerve function for pain relief, resulting in a functional deficit, and likely is less effective at the prevention of neuroma recurrence than active methods of the surgical reconstruction as described in Eberlin and Ducic’s work on this topic [11]. Active methods of surgical correction are more complex and time-consuming procedures that require the expertise of a nerve surgery specialist. It is for this reason that a simpler, less time-consuming method for neuroma and neurostenalgia prevention that can be undertaken at the primary surgery is of great importance in this field.

Neuromas and neurostenalgia following SRN repair cause significant morbidity to affected patients, with research indicating that the presence of chronic nerve pain can significantly impact a patient’s overall quality of life and independent function [12]. Thus far, techniques have only been described to treat these issues after they arise and are plagued with recurrence and treatment failure in this specific nerve. Newer techniques have begun looking at protective barriers to protect injured nerves at the time of revision surgery. The options include autologous tissue, allograft tissue, composite, and artificial materials.

Artificial and biological substitutes aim to use synthetic or collagen matrices to provide support and prevent external tethering. However, they are expensive, can inhibit neovascularisation, and potentially cause oedema and fibrosis [9,13]. Various autologous tissues have been studied as an adjunct to neurolysis. Veins have shown some promise as the smooth intima has been shown to improve nerve gliding, preventing scarring between the epineurium and the vein intima [14,15]. Unwrapped nerves in animal models with histologic sections showed marked degeneration and surrounding scar tissue, fewer axons, and more fibrous connective tissue. Nerves with a vein wrap had more axons, less degeneration, and less demyelination. Post-operative motor and sensory nerve conduction velocities following vein wrapping of scarred nerves show improvement compared to pre-operative studies [14]. Additionally, there is less latency in scarred nerves wrapped with veins compared to those that were not wrapped in electro-diagnostic studies [15]. Evidence shows improved pain scores, improved two-point discrimination, and improved grip strength [14]. The success rate in reducing pain scores using a vein wrap was 77% in one cohort of lower limb patients [16]. However, vein wraps require a donor site, sufficient length of vein, and increased surgical time to split and wrap the nerve, as well as multiple sutures to secure the vein as it spirals along the nerve. These multiple sutures along the nerve serve as a potential cause of nerve irritation.

The mechanisms thought to provide the clinical benefit of autologous flaps include improved nutrition from a highly vascularised flap bed, improved padding from external forces, separation of nerve from movement of adjacent tissue, superior gliding within the flap itself, physical restriction of axonal growth into surrounding tissue, and restriction of scar adhesion [5-7,17]. Protecting a nerve with a vascularised flap following nerve repair has been shown to improve regenerative outcomes. In one meta-analysis of 14 studies (n = 294), the use of flap coverage during revision carpal tunnel release led to an 86% success rate, compared to 74% in decompression alone [17].

Animal models have shown that wrapping adipofascial conduits around a site of primary neurorrhaphy reduces scar formation, with histological evidence of better recovery to original fibre diameter and myelin sheath thickness, and that in-setting adipose tissue around sites of nerve damage can promote neuronal regeneration and functional recovery [6]. Such findings have been replicated in clinical studies looking at neuroma treatment. Patients receiving well-vascularised soft tissue coverage for painful neuromas and chronically scarred nerves show significant improvement and symptom resolution. One case study of note by Yamamoto et al. recruited a patient similar to our patient cohort [7]. The patient sustained wrist trauma from a chainsaw injury and underwent SRN repair, following which he developed persistent nerve pain. This was initially treated with neurolysis but recurred four weeks later. The patient then underwent further neurolysis and coverage with a free temporoparietal fascial flap to prevent re-adhesion (local tissue could not be used due to extensive scar tissue formation in the forearm), and the authors noted significant improvement in pain during wrist and thumb motion [7].

The success of this technique employed in this one case report has been built upon by several larger studies. One such study by Krishnan et al. recruited seven patients with post-traumatic nerve injuries who had developed painful stump neuromas [5]. When these were covered with fasciocutaneous perforator flaps, five of the patients returned to their original professions, and all seven stopped taking opioids or requiring nerve stimulators [5]. In another case series of seven patients with recurrent median nerve neuropathy treated by revision surgery, Uemura et al. carefully and completely carried out neurolysis of the scarred median nerve using a distally based radial artery perforator adipose flap [18]. They recorded no recurrence of median nerve adhesion neuropathy and no major complications [18]. In another case series by Tham et al., six patients underwent re-exploration and neurolysis of the median nerve in the carpal tunnel for symptoms of recurrent median nerve compression and undertook an adjunctive reverse radial artery fascial flap procedure [19]. All patients had had at least two previous nerve decompressions but persistent symptoms. There was an improvement in all patients, with two patients describing the complete resolution of pain [19]. Previous studies have also demonstrated the successful use of radial or ulnar artery-based adipofascial flaps following neurolysis of the median nerve. Eight patients were treated by Adani et al. following post-traumatic painful median nerve neuromas [8]. In these patients, neurolysis and coverage with a radial or ulnar artery perforator adipofascial flap resulted in significant improvement in postoperative pain with complete resolution in five patients [8].

However, there are some issues with several of the flaps mentioned above: they are bulky, require thinning, and can cause donor site morbidity. One issue with many of the nerve-wrapping techniques previously described is that they require multiple sutures to hold the wrap in place, which introduces more foreign material, more inflammatory reaction, and more substance that needs to be broken down. Our technique uses minimal sutures that are also away from the nerve repair site, reducing the inflammatory burden. As there is abundant adipofascial tissue in the forearm and wrist, there seems to be little need for free flaps except in severely traumatised cases. Furthermore, no previous studies have looked at the use of an autologous flap as a prophylactic measure. Our novel surgical technique described above is inert, non-time-consuming, and simple to replicate by a non-nerve-specialist surgeon. Our hypothesis is that this procedure may work as a preventative measure rather than a cure, offering faster time to recovery following nerve injury and fewer trips to theatre with preservation of native nerve function.

Limitations

The limitations of the study were the small sample size and the retrospective nature that did not allow for comparative analysis. Further prospective studies are needed with a larger study group and increased post-operative surveillance with the use of validated outcome measures such as the QuickDASH patient-reported outcome measure, a pain score, and the presence of a static Tinel on clinical examination measured against a control group. Multivariate regression analysis that considers comorbidities, other injuries, and mechanisms of action would also be needed to assess causality. In this study, our follow-up data were limited to patients who did not attend clinic; however, we believe that we have captured an appropriate time frame of the post-operative recovery in these patients, as most recurrence of neural adhesions appears one to three months after trauma or surgery.

## Conclusions

In this technical report, we found that performing a double-opposing adipofascial interposition flap after SRN and adjacent tendon repairs was associated with no complications and no scar tethering or neuroma formation. An adipofascial flap may be a useful option for nerve protection after repair of the SRN, providing a gliding, interposing layer between not only the nerve and the other tendon repairs but also the nerve and the skin. This technique provides a simple and quick-to-perform option to insulate the SRN and prevent the recurrent complications associated with irritation and injury to this nerve. There are many studies looking at the management of neuromas and nerve scarring, but this is the first paper describing a primary preventative measure, and in the case of the SRN, prevention is better than cure.
